# Complications of Implantable Cardioverter Defibrillator and Their Potential Risk Factors in Patients with Hypertrophic Cardiomyopathy

**DOI:** 10.1155/2023/4552100

**Published:** 2023-11-13

**Authors:** Mohammad Taghi Hedayati Goudarzi, Maryam Moradi, Saeed Abrotan, Mehrdad Saravi, Hoda Shirafkan, Rana Irilouzadian, Hossein Salehi Omran

**Affiliations:** ^1^Cardiology Department, Rohani Hospital, School of Medicine, Babol University of Medical Sciences, Babol, Iran; ^2^Department of Cardiology, Faculty of Medicine, Babol University of Medical Sciences, Babol, Iran; ^3^Social Determinants for Health Research Center, Health Research Institute, Babol University of Medical Sciences, Babol, Iran; ^4^Burn Research Center, Iran University of Medical Sciences, Tehran, Iran

## Abstract

**Background:**

Hypertrophic cardiomyopathy (HCM) has different complications such as cardiac arrhythmia and sudden cardiac death (SCD). Insertion of an implantable cardioverter defibrillator (ICD) is recommended for HCM patients who are at high risk of SCD and malignant arrhythmias, despite having their own potential complications. *Hypothesis*. We aimed to investigate the prevalence of different complications of ICD insertion and the impact of the potential influential baseline characteristics in a one-year follow-up period.

**Methods:**

This was a retrospective study with a total of 71 HCM patients with ICD insertion. We evaluated the prevalence of different complications of ICD implantation and the impact of baseline characteristics on the occurrence of ICD complications using multivariate regression analysis in three 4-month periods.

**Results:**

In a one-year follow-up, 13 patients (18.3%) experienced at least one of the complications including pneumothorax, lead failure, ICD infection, inappropriate shocks, perforation, and upper limb deep vein thrombosis (DVT) with no mortality. Inappropriate shocks were reported as the most common (11.3%) complication during this period, with a gradual increase in the second (4.2%) and third (5.6%) follow-up sessions. Among all of the baseline characteristics that were investigated in this study, a positive history of hypertension was the only risk factor with significant impact on the occurrence of complications (*P* = 0.01).

**Conclusion:**

We demonstrated the occurrence of complications during a one-year follow-up as 18.3% in HCM patients with ICD insertion. A positive history of hypertension was the only baseline characteristic affecting the occurrence of complications, and inappropriate shocks were the most common complication.

## 1. Introduction

Hypertrophic cardiomyopathy (HCM) is a common inherited cardiac disorder that affects approximately one in every 500 individuals and accounts for one percent of all of the heart disease cases. It is characterized by abnormal thickening of the walls of the ventricles, which can lead to a variety of complications including heart failure, cardiac arrhythmias, and sudden cardiac death (SCD) [1, 2].

Prior studies had estimated the annual mortality rate of HCM to be between 3 and 6%; however, recent investigations have shown this rate to have decreased to less than 1% due to technological advancements, such as the insertion of an implantable cardioverter defibrillator (ICD) [3–6].

ICDs are often recommended for patients with HCM who are at high risk of SCD [7, 8]. However, despite their effectiveness in preventing SCD, ICDs are not free of their own potential complications, including inappropriate shock and device-related adverse events that can affect patients' quality of life and overall health outcomes [9–12].

According to the aforementioned elaborations, we aimed to investigate the prevalence of various complications resulting from ICD implantation in individuals with HCM, as well as to explore the potential risk factors that may have an impact on these complications in a retrospective cohort study.

## 2. Materials and Methods

We retrospectively analyzed data from 71 participants with HCM who underwent ICD insertion and were referred to Rohani Hospital in Babol from March 2011 to March 2021. Accordingly, they were investigated regarding the prevalence of different complications of ICD insertion and influential baseline characteristics during a one-year follow-up period divided into three equal intervals of four months. All HCM patients aged 18 or above who were referred to our hospital were included in this study, while nonhypertrophic cardiomyopathies and the history of cardiac surgery were our exclusion criteria. An ICD device manufactured by Medtronic (Medtronic Inc., Minneapolis, MN) was inserted for all of the participants. The diagnosis of HCM was based on the clinical presentations and echocardiography findings [1, 13, 14]. HCM was characterized by the presence of left ventricular hypertrophy (LVH) that cannot be explained by other potential causes like hypertension or infiltrative disorders. The hypertrophy associated with HCM is often asymmetric and typically involves the septum. A maximal wall thickness of 15 mm is commonly used as a cut-off point for diagnosis. In addition, a septal to posterior wall ratio of 1.3 or greater, as determined by imaging modalities such as echocardiography, computed tomography (CT), or cardiovascular magnetic resonance (CMR), is strongly indicative of HCM [15, 16]. After confirmation by imaging modalities, further history, electrocardiography (ECG), and laboratory tests were taken. In addition, baseline characteristics including age, height, weight, and clinical findings such as ICD implantation indications and sudden cardiac arrest (SCA) risk classification were investigated.

The complications associated with ICD insertion were categorized into two groups: those related to the implantation itself and those not related to the implantation. We examined complications such as hematoma, perforation, pneumothorax, deep vein thrombosis (DVT) of the upper limb, ICD lead failure, and lead infection as implantation-related complications. In addition, we investigated inappropriate shocks as nonimplantation-related complications. The necessary treatment measures were implemented accordingly. ICD inappropriate shocks were defined as cardiac shocks that occurred, following atrial fibrillation, sinus tachycardia, or device defect. Conversely, appropriate shocks were those that happened following ventricular fibrillation (VF) with a duration of less than 220 milliseconds or sustained VT (more than 30 beats). ICD lead failure was detected using an electrocardiogram performed during each follow-up session. Furthermore, an echocardiography was performed at each follow-up time to measure the left ventricular ejection fraction (LVEF).

Subsequently, we examined the influence of various baseline characteristics on the occurrence of complications following ICD insertion. These characteristics included gender, age, history of diabetes mellitus (DM), and hypertension, as well as the use of antiarrhythmic drugs. In terms of hypertension history, we considered all patients, including those with controlled and uncontrolled hypertension, as well as those with DM. The information regarding both hypertension and DM was obtained through self-reporting by the patients.

Sampling was taken using the convenience method with the sample size determined based on the possibility of 3.4% annualized ICD complications reported by Schinkel et al. [9], resulting in a minimum of 51 participants required for this study. This study was conducted by an approved ethical number of IR.MUMS.NURSE.REC.1400.083 from Babol University of Medical Sciences. In addition, written informed consent was obtained from all participants involved in this study.

### 2.1. Statistical Analysis

We analyzed data using the IBM SPSS Statistics for Windows, version 23 (IBM Corp, Armonk, NY, USA). The *p* value <0.05 was considered statistically significant. We used mean and standard deviation (SD) to express the quantitative data and frequency and percentage to express the qualitative data. We analyzed data using a *t*-test for quantitative variables and a chi-square test or Fisher's exact test for qualitative variables. In addition, logistic regression test was performed to examine the potential risk factors that may influence the complications of ICD insertion.

## 3. Results

**Figure 1:**
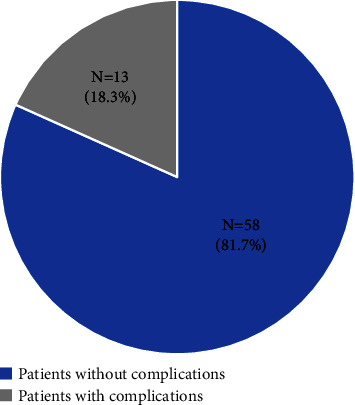
Prevalence of complications following ICD insertion in patients with hypertrophic cardiomyopathy in a one-year follow-up period.

A total of 71 participants with HCM were investigated in this study. The mean ± SD (range) age of the participants was 61.3 ± 15.0 (34–93) years, of whom 41 (57.7%) and 30 (42.3%) were male and female, respectively. A total of 58 (81.7%) participants had no complications during a one-year follow-up period, while the other 13 (18.3%) participants experienced some kind of complication following ICD insertion (Figure 1).

The baseline characteristics including age, gender, positive history of DM and hypertension, and antiarrhythmic drugs consumption were compared between the patients with and without complications. Hypertension was the only variable that showed a significant difference between the two groups (*P* = 0.03). A comparison of different variables between the two groups is presented in Table 1.

During the one-year follow-up period, we observed various complications related to ICD implantation, such as ICD lead failure, ICD infection, perforation, pneumothorax, and upper limb DVT. In addition, we observed inappropriate shocks as complications not directly related to ICD implantation. The prevalence of these complications in three different follow-up periods is shown in Table 2. During the first follow-up period, no complications related to ICD lead failure were reported. However, each of the other complications including pneumothorax, perforation, upper limb DVT, ICD infection, and inappropriate shock had the occurrence rate of 1.4%. In addition, the mean ± SD LVEF of patients with HCM was 52.2% ± 4.7% in this period of time.

In the second follow-up period, no complications in terms of pneumothorax, perforation, or lead infection were reported. A total of 3 (4.2%) patients experienced an inappropriate shock, and the occurrence of each upper limb DVT and lead failure was 1.4%. The mean ± SD LVEF of participants in the second follow-up period was 51.8% ± 4.4%.

During the third follow-up, none of the patients experienced complications of pneumothorax, perforation, upper limb DVT, lead infection, or lead failure. However, 4 (5.6%) patients experienced an inappropriate shock. The device was found to be functioning properly in 67 (94.4%) of the participants, and the mean ± SD LVEF was 51.7% ± 4.8%.

Noteworthy, no patient died during our one-year follow-up. Inappropriate shock was the most common complication (11.3%) during this period, which increased notably in the second and third follow-up sessions.

Logistic regression analysis (Table 3) demonstrated that hypertension was associated with an 8.5-fold increase in the risk of ICD implantation complications (*P* = 0.01). No significant influence was found for other variables investigated in this study.

## 4. Discussion

We aimed to estimate the prevalence of different complications resulting from ICD insertion in HCM patients and investigate the impact of potential influential risk factors over a one-year follow-up period. The prevalence of complications was estimated to be 18.3% in the total studied population during this time. Inappropriate shocks were found to be the most prevalent complication following ICD implantation with a prominent increase in the second (4.2%) and third (5.6%) 4-month follow-up sessions. Furthermore, hypertension was the only risk factor with a significant influence on the occurrence of complications in this study; no significant impact was observed for other variables mentioned in this manuscript.

HCM is a common inherited cardiac disorder affecting about one in every 500 individuals. This condition may lead to various adverse effects such as heart failure and SCD [1, 2]. Recent investigations have shown that the mortality rate of this disease has been decreased from 3-6% to less than 1% due to the use of technological advancemnts, such as ICD insertion [3–6]. However, it may have some potential complications including inappropriate shock and some device-related side effects such as perforation, pneumothorax, and lead infection [9–12]. In addition, these complications may be affected by a variety of risk factors such as the positive history of hypertension, DM, age, gender, and antiarrhythmic drug consumption which have been evaluated in this study.

Although few studies have investigated the prevalence of different complications of ICD insertion in HCM patients in other countries, this manuscript represents the first study conducted in Iran on this matter. In a study by Lin et al. [17], 181 participants with HCM were investigated regarding the prevalence of potential complications subsequent to ICD implantation during a mean follow-up of 59 months. Accordingly, 65 (36%) patients experienced some kind of complication during follow-up time. Similarly, they reported that inappropriate shocks were the most prevalent complication of ICD insertion. Furthermore, patients with atrial fibrillation and younger age were at higher risk of inappropriate discharges. In the present study, no significant impact of age was observed on the occurrence of complications. In addition, atrial fibrillation was not among the variables considered in our article.

In another retrospective study conducted by Syska et al. [10], a total of 104 HCM patients who underwent ICD insertion were investigated with a mean follow-up of 4.6 years. ICD complications consisted of inappropriate discharges (33.7%), lead failure (12.5%), and lead infections (4.8%) among participants during that period. In addition, four individuals died during the total follow-up time. Similar to our results, inappropriate shocks were the most prevalent complication reported during the follow-up period. In our article, no mortalities were reported, which could be attributed to the smaller sample size and shorter follow-up time compared to that study.

In another study, Vriesendorp et al. [11] included a total of 134 participants with HCM who were treated with ICDs. Subsequently, outcomes and complications following ICD insertion were investigated; 20 (14.9%) patients of the total participants experienced some kind of device-related complication during the follow-up period.

Ghavami et al. [18] performed a cross-sectional study involving 65 patients who underwent ICD implantation. They examined the impact of potential variables such as QRS duration before the procedure on the occurrence of inappropriate shocks following ICD insertion. No significant impact was observed between all the variables evaluated, except for the device manufacture. All ICDs implanted in our study were manufactured by Medtronic; hence, we were unable to assess the influence of device manufacture in this article. On the other hand, a positive history of hypertension, which had a significant effect on the prevalence of complications in our study, was not examined in their study.

In a single-center study by Frommeyer et al. [19], 18 HCM patients who were treated with subcutaneous ICD (S-ICD) were investigated regarding device complications with a follow-up duration of at least six months. Four participants (22%) experienced at least one episode of inappropriate discharge during follow-up time. This was indicative of an increased risk of inappropriate shocks following ICD insertion, similar to our study. The difference between our findings might be due to the smaller sample size of their study compared to ours.

In a cohort study conducted by Lambiase et al. [20], a total of 99 HCM patients and 773 non-HCM patients who underwent S-ICD were investigated over a mean follow-up time of 637 days. During the follow-up, 12.5% of the 99 HCM patients experienced an inappropriate shock which was similar to the findings in non-HCM patients (10.3%). These results are consistent with our findings.

Our study had some limitations, including a small sample size and a short follow-up period. Moreover, we only included a positive history of hypertension and DM as comorbidity disorders, potentially overlooking other comorbidities such as chronic kidney disorders, ischemic heart diseases, and dyslipidemia, which were not considered in this study. Nevertheless, there were also some strength points. To the best of our knowledge, this study was the first research carried out in our country concerning the prevalence of ICD complications and potential influential factors in HCM patients. In addition, we assessed the prevalence of various complications subsequent to ICD insertion at three different follow-up sessions, making our findings more precise and reliable compared to other studies conducted.

## 5. Conclusion

According to the findings of the present study, the prevalence of different complications of ICD insertion in patients with HCM was estimated to be 18.3% in a one-year follow-up time. In addition, inappropriate shocks were reported as the most prevalent adverse event observed during this period of time. Regarding the impact of different risk factors, a positive history of hypertension was the only variable that had a significant influence on the prevalence of ICD complications.

## Figures and Tables

**Table 1:** Comparison of different baseline characteristics between HCM patients with and without complications following the insertion of an implantable cardioverter defibrillator.

Variables		With complications	Without complications	*P* value
Gender	Male	7 (53.8%)	34 (58.6%)	0.75
	Female	6 (46.2%)	24 (41.4%)	

DM positive history	Yes	3 (23.1%)	19 (32.8%)	0.74
	No	10 (76.9%)	39 (67.2%)	

Hypertension positive history	Yes	9 (69.2%)	21 (36.2%)	0.03
	No	4 (30.8%)	37 (63.8%)	

Antiarrhythmic drugs consumption	Yes	7 (53.8%)	22 (37.9%)	0.29
	No	6 (46.2%)	36 (62.1%)	

Age, years		59.4 ± 16.2	61.7 ± 14.9	0.62

All data are expressed as the frequency and percentage for qualitative variables or the mean ± standard deviation for quantitative variables. Data analysis was performed using the chi-square test or Fisher's exact test for qualitative variables and *t*-test for quantitative variables. DM, diabetes mellitus; HCM, hypertrophic cardiomyopathy.

**Table 2:** Prevalence of different complications and LVEF following ICD insertion in a one-year follow-up divided into three equal parts.

Variables		First 4-month follow-up period	Second 4-month follow-up period	Third 4-month follow-up period	Total 1-year follow-up period
ICD implantation-related complications	Pneumothorax	1 (1.4%)	0 (0%)	0 (0%)	1 (1.4%)
	Perforation	1 (1.4%)	0 (0%)	0 (0%)	1 (1.4%)
	Upper limb DVT	1 (1.4%)	1 (1.4%)	0 (0%)	2 (2.8%)
	ICD infection	1 (1.4%)	0 (0%)	0 (0%)	1 (1.4%)
	Lead failure	0 (0%)	1 (1.4%)	0 (0%)	1 (1.4%)
	Total	4 (5.6%)	2 (2.8%)	0 (0%)	6 (8.5%)

ICD not implantation-related complications	Inappropriate shock	1 (1.4%)	3 (4.2%)	4 (5.6%)	8 (11.3%)

Total complications		5 (7.0%)	5 (7.0%)	4 (5.6%)	

LVEF, %		52.2 ± 4.7	51.8 ± 4.4	51.7 ± 4.8	

All of the data of the complications are expressed as the frequency and percentage for different complications of ICD insertion. LVEF is reported in the mean ± SD. DVT, deep vein thrombosis; ICD, implantable cardioverter defibrillator; LVEF, left ventricular ejection fraction.

**Table 3:** Multivariate analysis of the impact of different baseline characteristics on complication occurrence following ICD insertion.

Variables	OR	95.0% CI	*P* value
Gender	1.03	0.24–4.34	0.96
Age	0.96	0.91–1.01	0.17
DM positive history	0.30	0.05–1.53	0.15
Hypertension positive history	8.51	1.59-45.55	0.01
Antiarrhythmic drugs consumption	1.85	0.48–7.12	0.37

All data analyses were performed using multivariate regression test for all variables. CI, confidence interval; DM, diabetes mellitus; ICD, implantable cardioverter defibrillator; OR, odds ratio.

## Data Availability

The data that support the findings of this study are available upon reasonable request from the corresponding author.

## References

[1] Maron B. J., Gardin J. M., Flack J. M., Gidding S. S., Kurosaki T. T., Bild D. E. (1995). Prevalence of hypertrophic cardiomyopathy in a general population of young adults: echocardiographic analysis of 4111 subjects in the CARDIA study. *Circulation*.

[2] Maron B. J., Ommen S. R., Semsarian C., Spirito P., Olivotto I., Maron M. S. (2014). Hypertrophic cardiomyopathy: present and future, with translation into contemporary cardiovascular medicine. *Journal of the American College of Cardiology*.

[3] Elliott P. M., Gimeno J. R., Thaman R. (2005). Historical trends in reported survival rates in patients with hypertrophic cardiomyopathy. *Heart*.

[4] Maron B. J., Olivotto I., Spirito P. (2000). Epidemiology of hypertrophic cardiomyopathy–related death: revisited in a large non–referral-based patient population. *Circulation*.

[5] Aro A. L., Nair S. G., Reinier K. (2017). Population burden of sudden death associated with hypertrophic cardiomyopathy. *Circulation*.

[6] Hardarson T., Curiel R., De La Calzada C., Goodwin J. (1973). Prognosis and mortality of hypertrophic obstructive cardiomyopathy. *The Lancet*.

[7] Maron B. J., Shen W.-K., Link M. S. (2000). Efficacy of implantable cardioverter–defibrillators for the prevention of sudden death in patients with hypertrophic cardiomyopathy. *New England Journal of Medicine*.

[8] Maron B. J., Spirito P., Shen W.-K. (2007). Implantable cardioverter-defibrillators and prevention of sudden cardiac death in hypertrophic cardiomyopathy. *JAMA*.

[9] Schinkel A. F., Vriesendorp P. A., Sijbrands E. J., Jordaens L. J., ten Cate F. J., Michels M. (2012). Outcome and complications after implantable cardioverter defibrillator therapy in hypertrophic cardiomyopathy: systematic review and meta-analysis. *Circulation: Heart Failure*.

[10] Syska P., Przybylski A., Chojnowska L. (2010). Implantable cardioverter‐defibrillator in patients with hypertrophic cardiomyopathy: efficacy and complications of the therapy in long‐term follow‐up. *Journal of Cardiovascular Electrophysiology*.

[11] Vriesendorp P. A., Schinkel A. F., Van Cleemput J. (2013). Implantable cardioverter-defibrillators in hypertrophic cardiomyopathy: patient outcomes, rate of appropriate and inappropriate interventions, and complications. *American Heart Journal*.

[12] Wang N., Xie A., Tjahjono R. (2017). Implantable cardioverter defibrillator therapy in hypertrophic cardiomyopathy: an updated systematic review and meta-analysis of outcomes and complications. *Annals of Cardiothoracic Surgery*.

[13] Richardson P., McKenna W., Bristow M. (1996). Report of the 1995 world health organization/international society and federation of Cardiology task force on the definition and classification of cardiomyopathies. *Circulation*.

[14] Shapiro L. M., McKenna W. J. (1983). Distribution of left ventricular hypertrophy in hypertrophic cardiomyopathy: a two-dimensional echocardiographic study. *Journal of the American College of Cardiology*.

[15] Authors/Task Force members, Elliott P. M., Anastasakis A. (2014). 2014 ESC guidelines on diagnosis and management of hypertrophic cardiomyopathy: the task force for the diagnosis and management of hypertrophic cardiomyopathy of the European society of Cardiology (ESC). *European Heart Journal*.

[16] Henry W. L., Clark C. E., Glancy D. L., Epstein S. E. (1973). Echocardiographic measurement of the left ventricular outflow gradient in idiopathic hypertrophic subaortic stenosis. *New England Journal of Medicine*.

[17] Lin G., Nishimura R. A., Gersh B. J. (2009). Device complications and inappropriate implantable cardioverter defibrillator shocks in patients with hypertrophic cardiomyopathy. *Heart*.

[18] Ghavami Ghanbarabadi V., Jamali J., Heidari-Bakavoli A., Tayyebi M., Nazari Hyanlo H., Shakeri M. T. (2013). Factors influencing the status of appropriate and inappropriate shocks delivered by an implantable cardioverter defibrillator. *Journal of Birjand University of Medical Sciences*.

[19] Frommeyer G., Dechering D. G., Zumhagen S. (2016). Long-term follow-up of subcutaneous ICD systems in patients with hypertrophic cardiomyopathy: a single-center experience. *Clinical Research in Cardiology*.

[20] Lambiase P. D., Gold M. R., Hood M. (2016). Evaluation of subcutaneous ICD early performance in hypertrophic cardiomyopathy from the pooled EFFORTLESS and IDE cohorts. *Heart Rhythm*.

